# Association between oral microbiome alpha and beta diversity and MASLD risk: a large-scale, population-based retrospective study

**DOI:** 10.3389/fcimb.2026.1784034

**Published:** 2026-06-02

**Authors:** Zeru Liu, Yizhi Mao, Lei Zhang, Beibei Wang, Jiaxuan Tian, Yanjun Li, Shuangshuang Li, Yinglong He, Meiyan Zeng, Pan Meng, Houpan Song

**Affiliations:** 1 Hunan Provincial Key Laboratory of Traditional Chinese Medicine Diagnostics, Hunan University of Chinese Medicine, Changsha, Hunan, China; 2 School of Traditional Chinese Medicine, Hunan University of Chinese Medicine, Changsha, Hunan, China; 3 Changsha Hospital of Traditional Chinese Medicine, Changsha, Hunan, China; 4 School of Integrated Chinese and Western Medicine, Hunan University of Chinese Medicine, Changsha, Hunan, China

**Keywords:** cross-sectional study, gut-liver axis, metabolic dysfunction-associated steatotic liver disease, NHANES, oral microbiome diversity

## Abstract

**Background:**

Metabolic dysfunction-associated steatotic liver disease (MASLD) is the most common chronic liver disease globally, yet its pathogenesis remains incompletely understood. The “oral–gut–liver axis” hypothesis suggests that oral microbiota may influence liver metabolism through direct or indirect pathways; however, large-scale population-based evidence is still limited.

**Methods:**

Data from the 2009 to 2012 National Health and Nutrition Examination Survey (NHANES) included 2,759 U.S. adults aged ≥ 20 years. MASLD was defined using a U.S. Fatty Liver Index score ≥ 30. Oral rinse samples were sequenced targeting the 16S rRNA V4 region to evaluate alpha diversity (Observed OTUs, Faith’s Phylogenetic Diversity, Shannon-Wiener Index, and Inverse Simpson Index) and beta diversity (Bray-Curtis dissimilarity and UniFrac distance). Survey-weighted multivariable logistic regression models with sequential adjustment for demographic, lifestyle, and clinical metabolic covariates evaluated the association between oral microbial diversity and MASLD. Analyses were stratified by body mass index and smoking status.

**Results:**

The final analysis included 2,759 adults, of whom 183 individuals had MASLD. Oral microbial richness and diversity were significantly lower in individuals with MASLD. Multivariable analyses demonstrated a strong inverse association between oral microbial diversity and MASLD risk: Each increase in diversity was associated with a substantially reduced likelihood of MASLD. A clear dose-response relationship was observed, with individuals in the highest bacterial diversity group having a 65% lower risk than those in the lowest group. This association remained significant after adjusting for age, body weight, and diabetes. Stratified analysis revealed that the association was consistent across different body weight groups but was modified by smoking status. Finally, we identified that the overall makeup of the bacterial communities in the mouth was distinctly different between individuals with and without MASLD.

**Conclusion:**

This study demonstrates the association between oral bacteria and liver disorders. We found that lower diversity of oral microbes is independently correlated with a higher risk of disease, even after accounting for factors such as weight and blood sugar. The protective role of a diverse oral microbiome can be reduced by smoking and increased body weight. These findings establish the oral microbiome as a new and independent factor in liver health.

## Introduction

Metabolic dysfunction-associated steatotic liver disease (MASLD) is the most prevalent chronic liver disease worldwide. It was previously known as non-alcoholic fatty liver disease or metabolic-associated fatty liver disease (Abdelhameed et al., 2024). Driven by the rising global prevalence of obesity and type 2 diabetes, MASLD affects approximately 30% of adults globally, accounting for an estimated 1.66 billion cases in 2019. Over the past three decades, its global prevalence has increased steadily from 17.6% in 1990 to 23.4% in 2019, representing an average annual increase of about 1.0%. In North America, the prevalence is approximately 31.2%, with particularly rapid growth among younger populations, placing a substantial and increasing burden on public health systems (Fouad et al., 2024; Miao et al., 2024). MASLD encompasses a broad pathological spectrum, including simple steatosis, non-alcoholic steatohepatitis, hepatic fibrosis, and progression to hepatocellular carcinoma. Despite its significant clinical impact, the precise pathogenesis of MASLD remains incompletely understood. The widely accepted “multiple-hit” pathogenesis model proposes that disease progression results from the combined effects of insulin resistance, disrupted lipid metabolism, oxidative stress, mitochondrial dysfunction, genetic and epigenetic alterations, and intestinal dysbiosis (Guo et al., 2022; Li et al., 2024; Phoolchund and Khakoo, 2024).

Over the past decade, the role of gut microbiota in the pathogenesis of MASLD has been increasingly well characterized (Portincasa et al., 2024). Gut dysbiosis contributes to disease progression by impairing intestinal barrier integrity, promoting the translocation of microbial metabolites (endotoxin and ethanol), dysregulating bile acid and short-chain fatty acid signaling, and activating the hepatic Toll-like receptor-NF-κB inflammatory axis. Together, these processes exacerbate intrahepatic lipid accumulation and fibrosis (Long et al., 2024; Schwärzler et al., 2024; Yong et al., 2024). In contrast, the potential influence of the oral cavity—the second largest microbial ecosystem in the human body—on MASLD has long been underappreciated. Recent studies have introduced the concept of the ‘‘oral-gut-liver axis,’’ proposing that oral microbiota affect hepatic metabolism through two primary pathways: (1) oral pathogens and their virulence factors enter the bloodstream directly and reach the liver through the portal or systemic circulation; (2) swallowed oral microbes translocate to the gastrointestinal tract, where they alter the gut microbiota composition and promote systemic low-grade inflammation and metabolic dysregulation (Imai et al., 2021; Albuquerque-Souza and Sahingur, 2022; Chen et al., 2023).

Multiple cross-sectional studies have demonstrated that patients with severe periodontitis display a significantly higher prevalence of MASLD (Aguiar et al., 2023; Sato et al., 2025). Additionally, crucial periodontal parameters, including probing depth and clinical attachment loss, are positively correlated with the severity of hepatic steatosis and fibrosis (Storjord et al., 2022). Further supporting this association, patients with MASLD exhibit distinct oral microbial dysbiosis, marked by reduced alpha diversity, an increased *Firmicutes/Bacteroidetes* ratio, and a significant enrichment of potential pathogens, including *Porphyromonas gingivalis*, *Aggregatibacter actinomycetemcomitans*, and *Fusobacterium nucleatum* (Rinčić et al., 2022). Notably, serum IgG antibody titers against these pathogens are positively associated with increased aminotransferase levels and advanced liver fibrosis scores (Nalkurthi et al., 2022).

Critically, the oral microbiota can reshape the distal microbial ecosystem through the “oral-gut” axis. In periodontitis, the swallowing of saliva introduces a large number of oral pathogens into the gastrointestinal tract. These microbes suppress beneficial genera, including *Bifidobacterium* and *Lactobacillus*, while enriching opportunistic pathogens such as *Escherichia coli* and *Klebsiella pneumoniae* (Lu et al., 2022). This dysbiosis contributes to impaired intestinal barrier function, systemic endotoxemia, and disordered bile acid metabolism, collectively exacerbating hepatic inflammation and steatosis (Demir and Tacke, 2022). Importantly, fecal microbiota transplantation experiments have revealed that transferring gut microbiota from *Porphyromonas gingivalis*-exposed mice into germ-free recipients is sufficient to reproduce MASLD-like pathology in the recipients, offering direct evidence for a causal role of the oral-gut-liver axis (Jia et al., 2024).

With the oral microbiome-MASLD association being increasingly recognized, the objective of this study was to determine this relationship by employing the National Health and Nutrition Examination Survey (NHANES) database, an internationally recognized cohort. We analyzed large-scale, population-based data from NHANES to assess the correlation between oral microbiome alpha and beta diversity and MASLD.

## Materials and methods

### Study design and participants

NHANES monitors the health and nutritional status of the U.S. civilian, non-institutionalized population. It uses a complex, multi-stage probability sampling design, with data released in two-year cycles. This cross-sectional study used data from 2009 to 2012 and included participants aged ≥ 20 years. The study population was classified into MASLD and non-MASLD groups according to predefined criteria. All data used in this study were obtained from the official website of NHANES (https://www.cdc.gov/nchs/nhanes/). The data collection involves computer-assisted personal interviews conducted in participants’ homes, followed by comprehensive physical examinations and biospecimen collection (blood and urine) at mobile examination centers. Our analysis pooled data from the 2009–2010 and 2011–2012 cycles, which had unweighted Mobile Examination Center response rates of 77.3% and 69.5%, respectively. The final study population consisted of individuals with complete data on oral microbiome profiles, MASLD status, and key covariates from the MEC component of the 2009–2012 surveys.

### Assessment of the oral microbiome

Detailed protocols for oral microbiome data collection in NHANES are available on the official website. After the collection of mouthwash samples and subsequent DNA extraction, sequencing was conducted by the Knight Laboratory at the University of California, San Diego. The V4 region of the 16S rRNA gene was amplified by polymerase chain reaction and sequenced. The resulting raw sequencing data were processed employing the Quantitative Insights Into Microbial Ecology (QIIME) 1 pipeline. Amplicon sequence variants were generated from forward and reverse FASTQ files using the Divisive Amplicon Denoising Algorithm 2 (DADA2) algorithm, which infers true biological sequences while correcting for sequencing errors. This analysis identified a total of 2,750 operational taxonomic units (OTUs). Taxonomic classification was performed across the following hierarchical levels: kingdom, phylum, class, order, family, genus, and species.

Microbial diversity was evaluated using both alpha and beta diversity metrics. Alpha diversity describes species diversity within individual samples and reflects species richness and/or evenness. In contrast, beta diversity quantifies the compositional differences in microbiome profiles between different ecosystems (Parikh et al., 2020). Together, these metrics help elucidate the microbial heterogeneity within and across samples.

**Figure 1:**
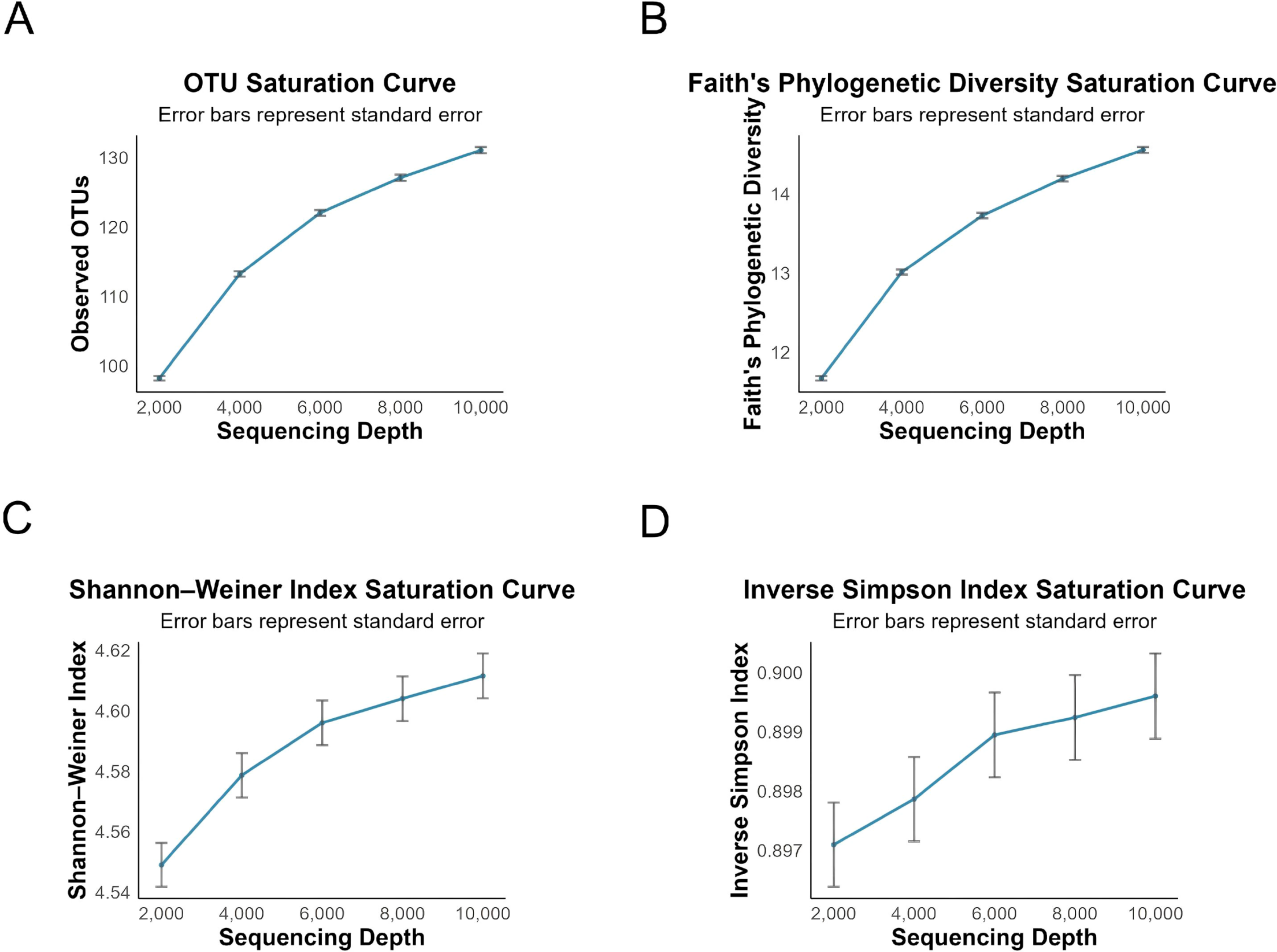
Saturation curves of oral microbiota alpha diversity indices across sequencing depths. The curves depict the mean values and standard errors of **(A)** OTUs, **(B)** Faith's phylogenetic diversity, **(C)** Shannon-Wiener index, and **(D)** Inverse Simpson index.

Four metrics were used to evaluate alpha diversity: OTU richness, the Shannon–Wiener index (SWI), the inverse Simpson index, and Faith’s phylogenetic diversity (FPD). Among these, OTU richness and FPD specifically measure species richness. In contrast, the SWI and inverse Simpson index combine species richness and evenness into a single composite measure. To ensure comparability across samples with varying sequencing depths, all alpha diversity analyses were based on a rarefaction depth of 10,000 sequences per sample (**Figure 1**). This threshold was examined as the point beyond which diversity estimates stabilized. The final alpha diversity value for each participant was calculated as the mean from 10 repeated rarefactions at this depth.

Beta diversity was evaluated using two complementary measures: Bray–Curtis dissimilarity and UniFrac distance. Bray–Curtis dissimilarity quantifies compositional differences between microbial communities based on OTU abundances. In contrast, UniFrac distance incorporates phylogenetic relationships to assess community divergence. Specifically, unweighted UniFrac distance considers only the presence or absence of lineages (unique branch lengths in the phylogenetic tree), whereas weighted UniFrac distance additionally accounts for relative species abundance by weighting branches according to OTU abundance differences (Chaturvedi et al., 2025).

### Definition of MASLD

Hepatic steatosis was expressed using the U.S. Fatty Liver Index (US-FLI), calculated as:


$$\text{US}-\text{FLI}=\frac{{\text{e}}^{\text{y}}}{1+{\text{e}}^{\text{y}}}\times 100$$

where *y = −0.8073 × non-Hispanic Black + 0.3458 × Mexican American + 0.0093 × age + 0.6151 × log_e_ (gamma-glutamyltransferase) + 0.0249 × waist circumference + 1.1792 × log_e_ (insulin) + 0.8242 × log_e_ (glucose) − 14.7812*. The variables “non-Hispanic Black” and “Mexican American” were assigned a value of 1 if the participant belonged to that respective ethnic group, and zero otherwise. A US-FLI score ≥ 30 was used as the diagnostic cutoff for fatty liver.

MASLD was characterized by a US-FLI score ≥ 30 in the absence of other known causes of chronic liver disorder. Specifically, participants were excluded if they had:

(1) viral hepatitis, as indicated by positive serological markers for hepatitis C or hepatitis B (Zhang et al., 2024); (2) significant alcohol consumption, defined as > 30 g/day for men and > 20 g/day for women (Li et al., 2020).

### Assessment of covariates

Participant characteristics and potential confounders were expressed as follows. Age was categorized into three groups: 20–39, 40–59, and ≥ 60 years. Race/ethnicity was classified as Mexican American, non-Hispanic White, non-Hispanic Black, other Hispanic, and other. Educational attainment was grouped into less than high school, high school graduate or equivalent General Educational Development (GED), and college or above. The poverty-income ratio (PIR) was stratified into four levels:< 1.0, 1.0–1.3, 1.4–3.0, and > 3.0. Hypertension was defined as a self-reported diagnosis and/or current use of antihypertensive medication, and diabetes mellitus was defined similarly based on self-reported diagnosis and/or hypoglycemic medication use. Smoking status was categorized as never (lifetime consumption of< 100 cigarettes), current (≥ 100 cigarettes in lifetime and currently smoking), and former (≥ 100 cigarettes in lifetime but having quit). Body mass index (BMI) was classified as ≤ 25.0, 25.0–29.9, and ≥ 30.0 kg/m^2^, corresponding to normal weight, overweight, and obesity, respectively. Dental treatment history was dichotomized as yes or no based on self-report. Laboratory values for alanine aminotransferase (ALT), aspartate aminotransferase (AST), and triglycerides (TG) were obtained directly from standard biochemical assays (Qiu et al., 2025).

### Statistical analysis

All statistical analyses accounted for the complex, multi-stage probability sampling plan of NHANES by incorporating sample weights, clustering, and stratification variables to ensure national representativeness and to correct for design effects. Sample weights were recalculated based on the NHANES analytical guidelines to align with the specific study sample. Analyses were performed using R software (version 4.5.0) with the ‘survey’ package. Continuous variables are presented as weighted means with standard errors, and group comparisons were conducted employing weighted linear regression models. Categorical variables are summarized as weighted percentages, and group comparisons were evaluated using the design-based Rao–Scott corrected chi-square test. A two-sided *P* < 0.05 was considered statistically significant.

For continuous variables, particularly the alpha diversity of the oral microbiome, distributions were first visually examined using density plots. This step was essential for evaluating the normality assumption underlying parametric tests, and for guiding the selection of appropriate descriptive statistics and the handling of variables in subsequent regression models. The association between oral microbial alpha diversity and MASLD risk was evaluated using multivariable logistic regression, with sequentially nested adjustments for potential confounders. Model 1 was an unadjusted crude model. Model 2 adjusted for demographic variables including age, sex, race/ethnicity, educational attainment, and the PIR. Model 3 was further adjusted for lifestyle and clinical covariates, including smoking status, hypertension, diabetes, BMI, dental treatment history, and ALT, AST, and TG levels. To visually depict differences in microbial community composition, principal coordinates analysis (PCoA) based on beta diversity was performed. Subgroup analyses and interaction tests were conducted across populations with different characteristics. Missing data were addressed using multiple imputation to preserve sample size and reduce potential selection bias (Qiu et al., 2025).

## Results

### Selection of the study population

**Figure 2:**
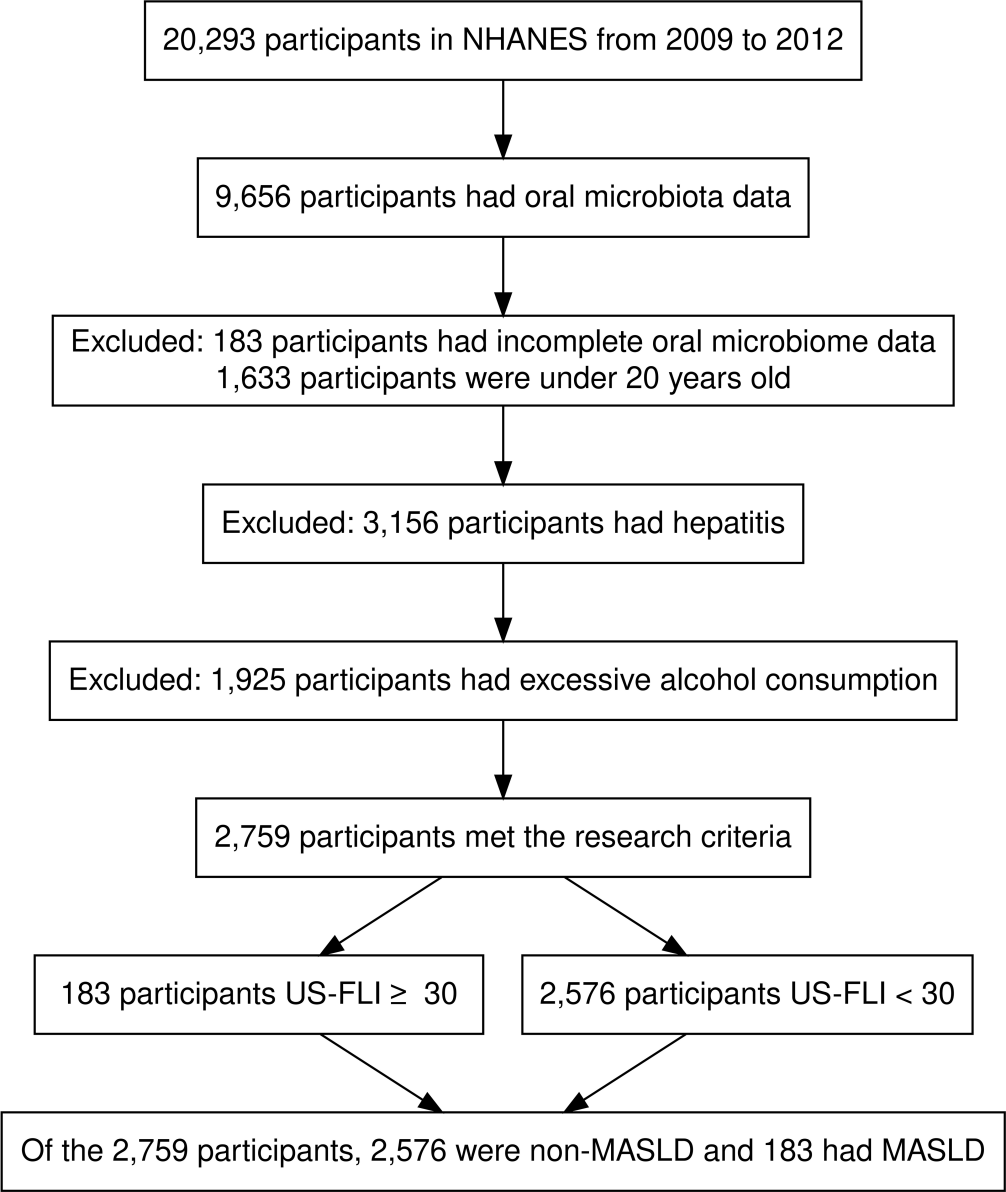
Flowchart illustrating the selection of the study population from the NHANES database.

Among the 20,293 participants in the NHANES database between 2009 and 2012, 9,660 had oral microbiome data available. From this initial pool, we sequentially excluded 187 participants with insufficient sequencing depth (< 10,000 sequences), 1,633 individuals younger than 20 years, and 5,081 participants with other potential causes of liver disorder, including significant alcohol consumption (> 30 g/day for men and > 20 g/day for women), a positive hepatitis B surface antigen, or positive hepatitis C antibody or RNA test. The final analytical sample comprised 183 participants with MASLD and 2,576 without MASLD. A detailed flowchart of the study population selection is provided in **Figure 2**.

### Baseline characteristics of study participants

**Figure 3:**
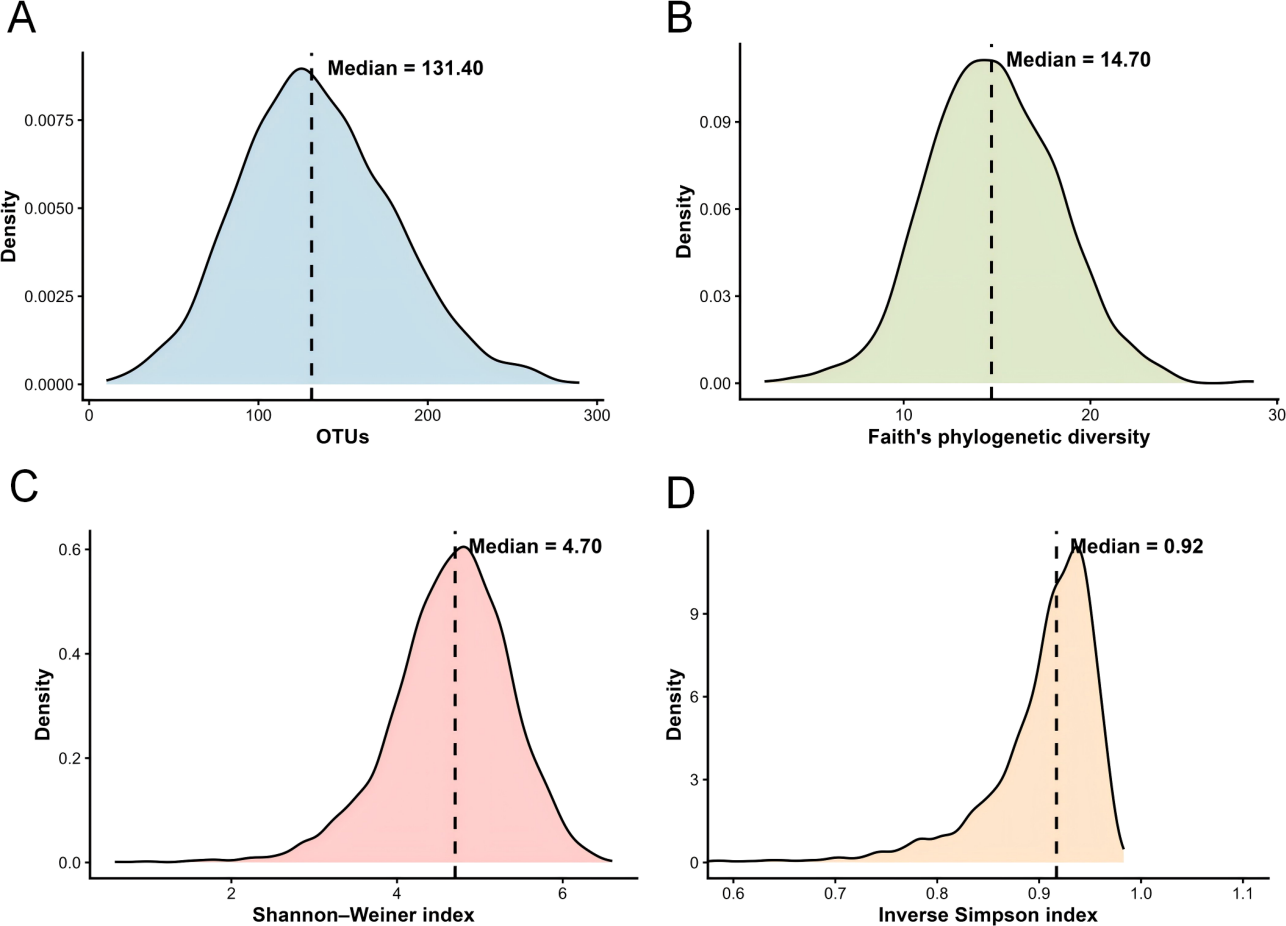
Distribution trends of four core alpha diversity indices of the oral microbiota: **(A)** OTUs, **(B)** Faith's phylogenetic diversity, **(C)** Shannon-Weiner index, and **(D)** Inverse Simpson index.

This research included 2,759 participants from the 2009 to 2012 NHANES cycles, with a mean age of 41.86 years. The cohort comprised 1,364 (49.43%) females and 1,395 (50.57%) males. Among them, 183 individuals were diagnosed with MASLD. The MASLD group exhibited a significantly higher mean age of 44.61 years. Significant differences (*P* < 0.05) were observed between MASLD and non-MASLD groups in terms of age, diabetes status, BMI, and serum levels of ALT, AST, and TG. Alpha diversity analysis demonstrated that all four oral microbiome indices were significantly lower in the MASLD group than in the non-MASLD group (*P* < 0.05). Detailed results are presented in **Table 1**, and **Figure 3** illustrates the differences across each diversity index.

**Table 1:** Weighted sample characteristics classified by MASLD status among U.S. adults.

Characteristic	MASLD Weighted N = 3,801,250 Unweighted N = 183	Non-MASLD Weighted N = 52,582,374 Unweighted N = 2,576	*P*-value
Age			0.042^2^
20-39	32.9%	46.2%	
40-59	55.9%	44.6%	
≥ 60	11.2%	9.2%	
Age (years)	45 (37, 54)	41 (31, 51)	0.006^3^
Gender, (%)			0.076^2^
Male	61.8%	51.5%	
Female	38.2%	48.5%	
Race/ethnicity, (%)			0.156^2^
Mexican American	12.7%	12.2%	
Non-Hispanic White	71.8%	64.4%	
Non-Hispanic Black	4.6%	10.9%	
Other Hispanic	6.0%	6.8%	
Other	4.8%	5.7%	
Education level, (%)			0.309^2^
< high school	22.8%	19.1%	
High school	28.1%	23.5%	
College	28.0%	28.8%	
> College	21.1%	28.6%	
Family income–poverty ratio, n (%)			0.443^2^
< 1	18.3%	19.7%	
1-1.3	11.8%	8.0%	
1.3-3.0	26.9%	28.7%	
≥ 3.0	43.1%	43.5%	
Diabetes, (%)			< 0.001^2^
YES	52.3%	20.9%	
NO	47.7%	79.1%	
Hypertension, (%)			0.064^2^
YES	85.7%	77.2%	
NO	14.3%	22.8%	
Smoke, (%)			0.268^1^
Current	19.1%	19.4%	
Former	25.9%	20.4%	
Never	55.0%	60.2%	
BMI, (%)			< 0.001^1^
≤ 25	8.3%	26.5%	
25-30	21.2%	33.1%	
≥ 30	70.5%	40.4%	
Oral treatment, (%)			0.779^1^
YES	13.2%	14.1%	
NO	86.8%	85.9%	
ALT, U/L	29 (23, 42)	21 (17, 29)	< 0.001^2^
AST, U/L	25 (21, 33)	23 (19, 27)	0.001^2^
TG, mg/dL	210 (142, 364)	120 (79, 186)	< 0.001^2^
OTUs	115 (95, 140)	126 (98, 157)	0.003^2^
Faith’s phylogenetic diversity	13.0 (11.3, 14.9)	14.1 (12.1, 16.6)	< 0.001^2^
Shannon–Wiener index	4.43 (4.10, 4.87)	4.65 (4.21, 5.07)	0.002^2^
Inverse Simpson index	0.91 (0.86, 0.93)	0.92 (0.88, 0.94)	0.028^2^

^1^Pearson’s χ^2^, Rao & Scott adjustment.

^2^Design-based Kruskal–Wallis test.

MASLD, metabolic dysfunction-associated steatotic liver disease; OTUs, operational taxonomic units; BMI, body mass index; ALT, alanine aminotransferase; AST, aspartate aminotransferase; TG: triglycerides.

### Association between oral microbiome alpha diversity and MASLD

To examine the association between MASLD and each of the four alpha diversity indices, separate analyses were conducted for each index. Multivariable logistic regression was applied with sequentially adjusted models to evaluate the influence of different sets of covariates. Model 1 provided an unadjusted estimate. Model 2 adjusted for key demographic and socioeconomic confounders, including age, sex, race/ethnicity, educational level, and the family income-poverty ratio. Model 3 further adjusted for clinical and metabolic covariates, including diabetes, hypertension, smoking status, BMI, dental treatment history, and serum ALT, AST, and TG levels. The consistency of the effect estimates across these models was evaluated to assess the robustness of the correlation between each diversity index and MASLD.

### Observed OTUs

When analyzed as a continuous variable, each unit increase in OTU count was correlated with a key reduction in MASLD risk in all models [odds ratio (OR) = 0.99, 95% confidence interval (CI): 0.99–1.00, *P* < 0.001]. A crucial dose–response association was evident in quartile analysis (with Q1 as the reference). In the fully adjusted Model 3, the OR for MASLD was 0.91 (95% CI: 0.59–1.39, *P* = 0.647) for Q2, 0.50 (95% CI: 0.31–0.81, *P* = 0.005) for Q3, and further reduced to 0.35 (95% CI: 0.20–0.59, *P* < 0.001) for Q4 (**Table 2**).

**Table 2:** OTUs and the MASLD logistic regression model.

Characteristic	Model 1			Model 2			Model 3		
	OR	95% CI	*P*-value	OR	95% CI	*P*-value	OR	95% CI	*P*-value
OTUs (continuous)	0.99	0.99, 1.00	< 0.001	0.99	0.99, 1.00	< 0.001	0.99	0.99, 1.00	< 0.001
OTUs									
Q1	—	—		—	—		—	—	
Q2	0.81	0.55, 1.19	0.292	0.84	0.57, 1.25	0.399	0.91	0.59, 1.39	0.647
Q3	0.56	0.37, 0.86	0.008	0.57	0.37, 0.89	0.013	0.50	0.31, 0.81	0.005
Q4	0.47	0.30, 0.73	< 0.001	0.45	0.28, 0.73	0.001	0.35	0.20, 0.59	< 0.001
*P* for trend			< 0.001			< 0.001			< 0.001

Model 1: no covariates were adjusted.

Model 2: adjusted for age (years); gender (%); race/ethnicity (%); education level (%); and family income-poverty ratio, n (%).

Model 3: adjusted for age (years); gender (%); race/ethnicity (%); education level (%); family income-poverty ratio, n (%); diabetes (%); hypertension (%); smoking status (%); BMI (%); oral treatment (%); ALT (U/L); AST (U/L); and TG (mg/dL).

### Faith’s phylogenetic diversity

As a continuous variable, each unit increase in Faith’s phylogenetic diversity was correlated with a significantly lower odds of MASLD in Model 1 (OR = 0.92, 95% CI: 0.88–0.96, *P* < 0.001), Model 2 (OR = 0.92, 95% CI: 0.88–0.96, *P* < 0.001), and Model 3 (OR = 0.90, 95% CI: 0.86–0.95, *P* < 0.001). Quartile analysis demonstrated a significant dose–response correlation. In the fully adjusted Model 3, compared to Q1, the ORs for MASLD were 0.85 (95% CI: 0.54–1.34, *P* = 0.481) for Q2, 0.43 (95% CI: 0.26–0.71, *P* = 0.001) for Q3, and 0.40 (95% CI: 0.24–0.67, *P* < 0.001) for Q4 (**Table 3**).

**Table 3:** Faith’s phylogenetic diversity and MASLD logistic regression model.

Characteristic	Model 1			Model 2			Model 3		
	OR	95% CI	*P*-value	OR	95% CI	*P*-value	OR	95% CI	*P*-value
Faith’s phylogenetic diversity (continuous)	0.92	0.88, 0.96	< 0.001	0.92	0.88, 0.96	< 0.001	0.90	0.86, 0.95	< 0.001
Faith’s phylogenetic diversity									
Q1	—	—		—	—		—	—	
Q2	0.80	0.54, 1.17	0.248	0.84	0.57, 1.25	0.399	0.86	0.56, 1.33	0.502
Q3	0.48	0.31, 0.74	< 0.001	0.50	0.32, 0.79	0.003	0.43	0.26, 0.71	0.001
Q4	0.52	0.34, 0.80	0.003	0.50	0.31, 0.79	0.003	0.40	0.24, 0.67	< 0.001
*P* for trend			< 0.001			< 0.001			< 0.001

Model 1: no covariates were adjusted.

Model 2: adjusted for age (years); gender (%); race/ethnicity (%); education level (%); and family income-poverty ratio, n (%).

Model 3: adjusted for age (years); gender (%); race/ethnicity (%); education level (%); family income-poverty ratio, n (%); diabetes (%); hypertension (%); smoking status (%); BMI (%); oral treatment (%); ALT (U/L); AST (U/L); and TG (mg/dL).

### Shannon–Wiener index

When treated as a continuous variable, each unit increase in the Shannon–Wiener index was significantly correlated with decreased odds of MASLD in Model 1 (OR = 0.69, 95% CI: 0.57–0.84, *P* < 0.001), Model 2 (OR = 0.70, 95% CI: 0.57–0.85, *P* < 0.001), and Model 3 (OR = 0.64, 95% CI: 0.51–0.80, *P* < 0.001). Quartile analysis indicated a significant inverse linear trend (*P*-trend ≤ 0.002 for all models). In the fully adjusted Model 3, compared to Q1, the OR for Q4 was 0.39 (95% CI: 0.23–0.67, *P* < 0.001), whereas non-significant correlations were observed for Q2 (OR = 1.08, 95% CI: 0.70–1.66, *P* = 0.738) or Q3 (OR = 0.95, 95% CI: 0.60–1.48, *P* = 0.814) (**Table 4**).

**Table 4:** Shannon–Wiener index and MASLD logistic regression model.

Characteristic	Model 1			Model 2			Model 3		
	OR	95% CI	*P*-value	OR	95% CI	*P*-value	OR	95% CI	*P*-value
Shannon–Wiener index (continuous)	0.69	0.57, 0.84	< 0.001	0.70	0.57, 0.85	< 0.001	0.64	0.51, 0.80	< 0.001
Shannon–Wiener index									
Q1	—	—		—	—		—	—	
Q2	0.94	0.64, 1.40	0.771	0.98	0.66, 1.47	0.941	1.08	0.70, 1.66	0.738
Q3	0.85	0.57, 1.26	0.415	0.86	0.57, 1.29	0.463	0.95	0.60, 1.48	0.814
Q4	0.44	0.27, 0.71	< 0.001	0.44	0.27, 0.72	0.001	0.39	0.23, 0.67	< 0.001
*P* for trend			0.001			0.002			0.002

Model 1: no covariates were adjusted.

Model 2: adjusted for age (years); gender (%); race/ethnicity (%); education level (%); and family income-poverty ratio, n (%).

Model 3: adjusted for age (years); gender (%); race/ethnicity (%); education level (%); family income-poverty ratio, n (%); diabetes (%); hypertension (%); smoking status (%); BMI (%); oral treatment (%); ALT (U/L); AST (U/L); and TG (mg/dL).

### Inverse Simpson index

When analyzed as a continuous variable, the Inverse Simpson index indicated non-significant correlation with MASLD risk in Model 1 (OR = 0.22, 95% CI: 0.03–1.68, *P* = 0.144) or Model 2 (OR = 0.21, 95% CI: 0.03–1.63, *P* = 0.134), but a significant inverse correlation emerged in the fully adjusted Model 3 (OR = 0.08, 95% CI: 0.01–0.76, *P* = 0.028). Quartile analysis demonstrated a significant dose–response relationship (*P*-trend ≤ 0.009 for all models). In Model 3, compared to Q1, the OR for Q4 was 0.52 (95% CI: 0.31–0.85, *P* = 0.010), while non-significant associations were found for Q2 (OR = 1.10, 95% CI: 0.71–1.69, *P* = 0.674) or Q3 (OR = 0.76, 95% CI: 0.48–1.21, *P* = 0.254) (**Table 5**).

**Table 5:** Inverse Simpson index and MASLD logistic regression model.

Characteristic	Model 1			Model 2			Model 3		
	OR	95% CI	*P*-value	OR	95% CI	*P*-value	OR	95% CI	*P*-value
Inverse Simpson index (continuous)	0.22	0.03, 1.68	0.144	0.21	0.03, 1.63	0.134	0.08	0.01, 0.76	0.028
Inverse Simpson index									
Q1	—	—		—	—		—	—	
Q2	1.06	0.72, 1.57	0.759	1.04	0.70, 1.55	0.830	1.10	0.71, 1.69	0.674
Q3	0.78	0.51, 1.18	0.243	0.76	0.50, 1.17	0.211	0.76	0.48, 1.21	0.254
Q4	0.58	0.37, 0.92	0.020	0.58	0.36, 0.92	0.020	0.52	0.31, 0.85	0.010
*P* for trend			0.009			0.009			0.004

Model 1, no covariates were adjusted.

Model 2, adjusted for age (years); gender (%); race/ethnicity (%); education level (%); and family income-poverty ratio, n (%).

Model 3, adjusted for age (years); gender (%); race/ethnicity (%); education level (%); family income-poverty ratio, n (%); diabetes (%); hypertension (%); smoking status (%); BMI (%); oral treatment (%); ALT (U/L); AST (U/L); and TG (mg/dL).

### Subgroup analysis of the association between oral microbiota alpha diversity and MASLD

#### Observed OTUs

In the BMI-stratified subgroup analysis, a key inverse correlation between OTUs and MASLD was observed across all BMI categories. The subgroups were as follows: BMI< 25 (n = 657, 23.8%), OR = 0.978 (95% CI: 0.963–0.994, *P* = 0.007); BMI 25–30 (n = 873, 31.6%), OR = 0.985 (95% CI: 0.976–0.995, *P* = 0.002); and BMI ≥ 30 (n = 1,229, 44.5%), OR = 0.994 (95% CI: 0.989–0.998, *P* = 0.004) (**Table 6**).

**Table 6:** Subgroup analysis on the correlation between MASLD and OTUs.

Subgroup	N (%)	OR (95% CI)	*P*	*P* for interaction
Age				0.3
20–39	1282 (46.5%)	1.00 (0.99-1.00)	0.003	
40–59	1141 (41.4%)	1.00 (0.99-1.00)	0.05	
≥ 60	336 (12.2%)	0.99 (0.98-1.00)	0.008	
Gender				0.44
Male	1395 (50.6%)	1.00 (0.99-1.00)	0.002	
Female	1364 (49.4%)	0.99 (0.98-1.00)	0.001	
Race ethnicity				0.15
Mexican American	597 (21.6%)	0.99 (0.99-1.00)	0.1	
Non-Hispanic White	1145 (41.5%)	0.99 (0.99-1.00)	0.05	
Non-Hispanic Black	501 (18.2%)	0.99(0.98-1.00)	0.14	
Other Hispanic	310 (11.2%)	0.98 (0.97-0.99)	0.003	
Other	206 (7.5%)	0.98 (0.96-0.99)	0.02	
Education level				0.69
< high school	763 (27.7%)	0.99 (0.99-1.00)	0.004	
High school	647 (23.5%)	0.99 (0.99-1.00)	0.09	
College	763 (27.7%)	0.99 (0.98-1.00)	0.002	
> College	581 (21.1%)	0.99 (0.98-1.00)	0.1	
Family income poverty ratio				0.49
< 1	817 (29.6%)	0.99 (0.98-0.99)	< 0.001	
1–1.3	342 (12.4%)	0.99 (0.99-1.00)	0.18	
1.3–3.0	786 (28.5%)	0.99 (0.99-1.00)	0.05	
≥ 3.0	814 (29.5%)	0.99 (0.98-1.00)	0.08	
Diabetes				0.82
YES	775 (28.1%)	0.99 (0.99-1.00)	0.001	
NO	1984 (71.9%)	0.99 (0.99-1.00)	0.002	
Hypertension				0.25
YES	2093 (75.9%)	0.99 (0.99-1.00)	< 0.001	
NO	666 (24.1%)	0.99 (0.98-1.00)	0.04	
Smoke				0.09
Current	582 (21.1%)	1.00 (0.99-1.00)	0.42	
Former	551 (20%)	0.99 (0.99-1.00)	0.002	
Never	1626 (58.9%)	0.99 (0.98-1.00)	< 0.001	
BMI				0.03
≤ 25	657 (23.8%)	0.963-0.994	0.007	
25–30	873 (31.6%)	0.976-0.995	0.002	
≥ 30	1229 (44.5%)	0.989-0.998	0.004	
Oral treatment				0.33
YES	473 (17.1%)	1.00 (0.99-1.01)	0.38	
NO	2286 (82.9%)	0.99 (0.99-1.00)	< 0.001	

#### Faith’s phylogenetic diversity

In the smoking status-stratified analysis, the sample sizes were as follows: current smokers (n = 582, 21.1%), former smokers (n = 551, 20.0%), and non-smokers (n = 1,626, 58.9%). Faith’s phylogenetic diversity was significantly correlated with MASLD risk in former smokers (OR = 0.86, 95% CI: 0.79–0.94, *P* = 0.001) and non-smokers (OR = 0.86, 95% CI: 0.79–0.93, *P* < 0.001), but not in current smokers (OR = 0.98, 95% CI: 0.90–1.06, *P* = 0.61). The interaction *P*-value for smoking status was 0.04.

In the BMI-stratified analysis, individuals were categorized as BMI ≤ 25 (23.9%), 25 to 30 (31.6%), and ≥ 30 (44.5%). A significant inverse correlation between Faith’s phylogenetic diversity and MASLD was observed in all BMI subgroups: BMI ≤ 25 (OR = 0.78, 95% CI: 0.65–0.93, *P* = 0.006); BMI 25 to 30 (OR = 0.84, 95% CI: 0.75–0.94, *P* = 0.002); and BMI ≥ 30 (OR = 0.92, 95% CI: 0.86–0.98, *P* = 0.005). The interaction *P*-value for BMI was 0.048 (**Table 7**).

**Table 7:** Subgroup analysis on the correlation between MASLD and Faith’s phylogenetic diversity.

Subgroup	N (%)	OR (95% CI)	*P*	*P* for interaction
Age				0.33
20–39	1282 (46.5%)	0.89 (0.82-0.97)	0.01	
40–59	1141 (41.4%)	0.93 (0.88-0.99)	0.03	
≥ 60	336 (12.2%)	0.82 (0.72-0.94)	0.003	
Gender				0.52
Male	1395 (50.6%)	0.91 (0.86-0.96)	0.001	
Female	1364 (49.4%)	0.88 (0.81-0.95)	0.001	
Race ethnicity				0.17
Mexican American	597 (21.6%)	0.93 (0.85-1.03)	0.15	
Non-Hispanic White	1145 (41.5%)	0.93 (0.87-1.00)	0.05	
Non-Hispanic Black	501 (18.2%)	0.86 (0.74-1.00)	0.05	
Other Hispanic	310 (11.2%)	0.76 (0.64-0.91)	0.002	
Other	206 (7.5%)	0.80 (0.65-0.99)	0.04	
Education level				0.63
< high school	763 (27.7%)	0.91 (0.85-0.96)	0.007	
High school	647 (23.5%)	0.92 (0.83-1.01)	0.09	
College	763 (27.7%)	0.86 (0.78-0.95)	0.002	
> College	581 (21.1%)	0.84 (0.71-0.99)	0.04	
Family income poverty ratio				0.75
< 1	817 (29.6%)	0.86 (0.80-0.93)	< 0.001	
1–1.3	342 (12.4%)	0.93 (0.84-1.04)	0.23	
1.3–3.0	786 (28.5%)	0.91 (0.84-1.00)	0.05	
≥ 3.0	814 (29.5%)	0.88 (0.79-0.98)	0.02	
Diabetes				0.86
YES	775 (28.1%)	0.92 (0.84-0.96)	0.001	
NO	1984 (71.9%)	0.90 (0.84-0.96)	0.002	
Hypertension				0.25
YES	2093 (75.9%)	0.91 (0.86-0.96)	< 0.001	
NO	666 (24.1%)	0.88 (0.78-0.99)	0.04	
Smoke				0.04
Current	582 (21.1%)	0.98 (0.90-1.06)	0.61	
Former	551 (20%)	0.86 (0.79-0.94)	0.001	
Never	1626 (58.9%)	0.86 (0.79-0.93)	< 0.001	
BMI				0.048
≤ 25	657 (23.9%)	0.78 (0.65-0.93)	0.006	
25–30	873 (31.6%)	0.84 (0.75-0.94)	0.002	
≥ 30	1229 (44.5%)	0.92 (0.86-0.98)	0.005	
Oral treatment				0.24
YES	473 (17.1%)	0.96 (0.86-1.08)	0.54	
NO	2286 (82.9%)	0.89 (0.84-0.93)	< 0.001	

#### PCoA of oral microbiome beta diversity

**Figure 4:**
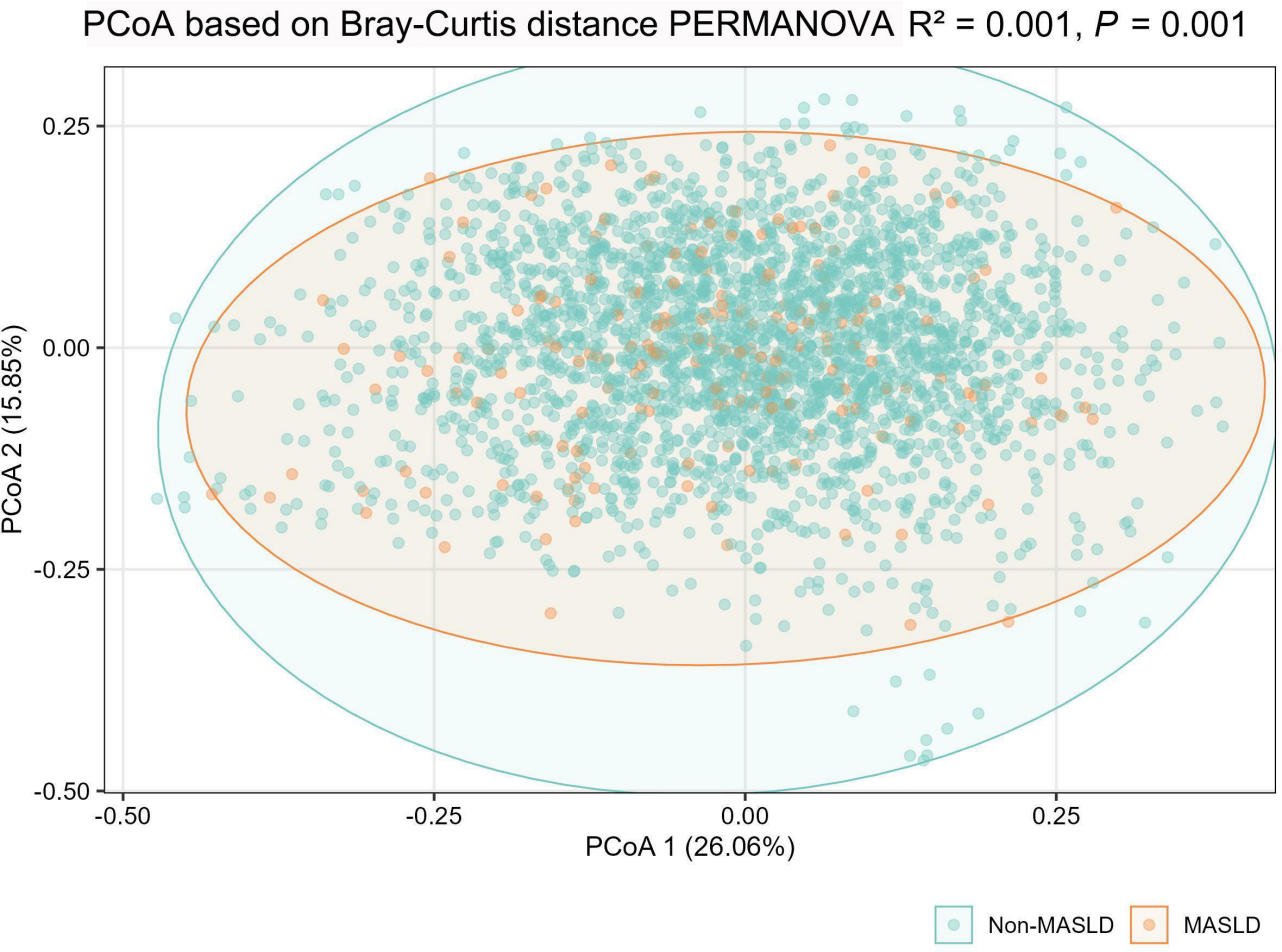
Principal coordinate analysis (PCoA) of oral microbiota beta diversity based on Bray-Curtis dissimilarity.

PCoA based on Bray–Curtis dissimilarity demonstrated that the first two principal coordinates accounted for 26.06% (PCo1) and 15.85% (PCo2) of the total variance, respectively. PERMANOVA testing identified a statistically significant, albeit small, difference in microbial community composition between groups (R^2^ = 0.001, *P* = 0.001) (**Figure 4**).

## Discussion

Leveraging the nationally representative NHANES dataset, this study utilized the US-FLI to identify 183 MASLD cases among 2,759 participants, providing a strong foundation for evaluating the oral microbiome-MASLD association. The MASLD group displayed a significantly higher mean age and marked differences in crucial metabolic parameters, including diabetes prevalence, BMI, ALT, AST, and TG levels, than the non-MASLD group. This metabolic profile aligns with the components of the US-FLI, affirming the cohort’s representativeness. The clinical relevance of these metabolic derangements is highlighted by recent evidence; for instance, increased liver enzymes in MASLD have been associated with poorer treatment responses, even in cases with ostensibly normal ALT levels (Luo et al., 2025). Furthermore, the systemic inflammatory state correlated with this metabolic phenotype is increasingly recognized as a key modifier of cardiovascular risk, thereby situating our results within a broader pathophysiological context (Wissel et al., 2025).

Our analysis demonstrated a significant reduction across all four alpha diversity indices in the MASLD group. This consistent pattern suggests a markedly impoverished oral microbiome ecology. Specifically, the decrease in observed OTUs and Faith’s phylogenetic diversity denotes a loss in species richness and evolutionary breadth, whereas the concurrent declines in the Shannon–Wiener and Inverse Simpson indices further indicate a disruption in community evenness. This collective erosion of diversity represents a less stable microbial environment in patients with MASLD, a result that aligns with emerging research associating oral dysbiosis to systemic metabolic conditions (Niu et al., 2023; Huang et al., 2024). The observed decline in ecological stability may not merely reflect an association but also compromised microbial resilience, which is increasingly recognized as a crucial factor in the pathogenesis of metabolic disorders (Frost et al., 2021; Watson et al., 2023; Moseeb et al., 2025).

Multivariable logistic regression identified a strong, inverse dose–response correlation between alpha diversity and MASLD risk. An increase in observed OTUs as a continuous variable was correlated with a reduction in MASLD odds—an effect significant at the population level. Quartile-based analysis reinforced this gradient; compared to Q1, the MASLD risk was lower in Q3 and Q4. A similar protective trend was observed for Faith’s phylogenetic diversity, with Q4 demonstrating a reduction in risk compared with Q1. Notably, a significant protective effect for the Shannon–Wiener index emerged only in Q4, indicating a potential threshold effect, in which only high diversity confers a measurable benefit. This pattern aligns with the concept of “microbial resilience,” in which a diverse microbiome may buffer against metabolic dysregulation until a crucial loss of diversity occurs (Huang et al., 2024; Sato et al., 2025). The identification of such a threshold could have implications for future microbiome-targeted interventions, indicating that restoring diversity beyond a threshold level may be necessary to achieve clinical impact in MASLD (Augustijn et al., 2025; Saeed et al., 2025).

Subgroup analyses yielded further nuance. When stratified by smoking status, Faith’s phylogenetic diversity indicated a non-significant association with MASLD among current smokers, whereas crucial protective effects were observed in both former and non-smokers. This indicates that active smoking may disrupt the functional crosstalk between the oral microbiome and hepatic metabolism. Similarly, BMI-stratified analyses demonstrated a gradient of effect attenuation with increasing adiposity. The inverse correlation was strongest in normal-weight individuals, moderated in the overweight group, and weakest, despite being still significant, in those with obesity. This pattern suggests that the potent drive of obesity in MASLD pathogenesis may partially overwhelm the independent contribution of microbial diversity, a phenomenon that aligns with the concept of an “obesity-dominated” metabolic state in which systemic inflammatory and metabolic dysregulation supersedes more subtle regulatory pathways (Varra et al., 2024; Moseeb et al., 2025; Xie et al., 2025). The observed interaction with smoking further underscores the effects of environmental exposures on the microbiome-MASLD axis, indicating that lifestyle interventions could modulate this association (Zhang et al., 2025b).

Beta diversity analysis demonstrated a significant overall separation in oral microbial community structure between MASLD and non-MASLD groups, as measured by Bray–Curtis dissimilarity, albeit with a modest effect size. This indicates that MASLD is correlated not only with decreased diversity but also with distinct compositional changes. The first two principal coordinates accounted for the variance, highlighting dominant structural patterns. These results indicate that MASLD may be associated with an ecological reorganization of the oral microbiota, a phenomenon increasingly reported in systemic metabolic conditions (Zhang et al., 2025a). Although the effect size is limited, such consistent structural shifts could reflect altered functional potential or specific pathogen enrichment, providing a plausible pathway associating oral ecology with hepatic health (Elghannam et al., 2023).

Using US-FLI as a non-invasive proxy for MASLD, which is distinct from imaging or histology, is grounded in routine clinical metrics of systemic metabolic status, thereby improving the biological plausibility of our key finding that oral microbiome alterations are correlated with systemic metabolic dysregulation. Crucially, even after rigorously adjusting for essential metabolic confounders, including diabetes, hypertension, BMI, and liver enzymes, which are themselves US-FLI components, the significant microbiome-MASLD association persisted. This suggests that the oral microbiome influences MASLD risk through pathways at least partially independent of conventional metabolic factors. Mounting evidence indicates that such independence may increase from direct mechanisms, including immune modulation by bacterial antigens or the systemic dissemination of orally derived metabolites (Li et al., 2025). Recent studies specifically implicate bacterially derived short-chain fatty acids and secondary bile acids in modulating hepatic inflammation and lipid metabolism, offering a plausible mechanistic basis for the complementary risk pathway demonstrated by our statistical analysis (Niu et al., 2025; Zhong et al., 2025).

Several limitations should be acknowledged. First, the cross-sectional design of the NHANES 2009–2012 survey may preclude establishing causality between oral dysbiosis and MASLD. Although our statistical models positioned oral microbial diversity as an independent predictor based on the ‘‘oral-gut-liver axis’’ hypothesis (Imai et al., 2021), the biological relationship is likely bidirectional. Systemic metabolic and immunological disruptions inherent in MASLD could secondarily alter the oral microenvironment. Therefore, the exact temporal sequence requires clarification through prospective longitudinal cohorts. Second, despite adjusting for key lifestyle variables and self-reported dental history, we lacked detailed clinical dental records—such as exact tooth count, precise occlusion, and specific periodontal parameters—which are known to strongly influence oral microbiota composition (Balan et al., 2025). Fine-grained dietary data were also unavailable. Finally, while standard metabolic medications were accounted for, specific data regarding microbiome-altering drugs, notably recent antibiotic or proton pump inhibitor use, could not be incorporated (Imhann et al., 2016). Consequently, residual confounding from these unmeasured clinical, dietary, and pharmacological factors cannot be excluded, underscoring the need for further investigations with more comprehensive phenotypic annotation.

## Conclusions

This study highlights a crucial and independent correlation between oral microbiome dysbiosis and MASLD. The results reveal that reduced alpha diversity, indicative of an ecologically impoverished and less resilient oral microbial community, displays a strong inverse dose–response correlation with MASLD risk. Concurrent differences in microbial community structure (beta diversity) further confirm that MASLD is associated with a distinct oral microbial ecology. Collectively, these findings suggest that the oral microbiome may serve as a new and complementary risk factor or biomarker in MASLD pathogenesis. Evidence of a potential diversity threshold and the modulatory effects of lifestyle factors provide a strong foundation for future research into microbiome-targeted diagnostics and interventions in MASLD.

## Data Availability

Publicly available datasets were analyzed in this study. This data can be found here: National Health and Nutrition Examination Survey (NHANES): https://www.cdc.gov/nchs/nhanes/.
