# Supplementary material for: Association between oral microbiome alpha and beta diversity and MASLD risk: a large-scale, population-based retrospective study
Source: Front Cell Infect Microbiol. 2026 Jun 2;16:1784034. doi: 10.3389/fcimb.2026.1784034 (PMC13268953; doi:10.3389/fcimb.2026.1784034)
Supplement: Supplementary file 1 [file Table1.docx]

**Supplementary Material**

**Supplementary Table 1.** Subgroup analysis on the correlation between MASLD and Shannon–Wiener Index.

| Subgroup | N (%) | OR (95% CI) | *P* | *P* for interaction |
| --- | --- | --- | --- | --- |
| Age |  |  |  | 0.81 |
| 20–39 | 1282 (46.5%) | 0.62 (0.43-0.9) | 0.01 |  |
| 40–59 | 1141 (41.4%) | 0.69 (0.51-0.93) | 0.01 |  |
| ≥ 60 | 336 (12.2%) | 0.56 (0.32-0.98) | 0.04 |  |
| Gender |  |  |  | 0.49 |
| Male | 1395 (50.6%) | 0.59 (0.45-0.79) | <0.001 |  |
| Female | 1364 (49.4%) | 0.69 (0.5-0.95) | 0.02 |  |
| Race ethnicity |  |  |  | 0.82 |
| Mexican American | 597 (21.6%) | 0.7 (0.44-1.12) | 0.14 |  |
| Non-Hispanic White | 1145 (41.5%) | 0.69 (0.51-0.92) | 0.01 |  |
| Non-Hispanic Black | 501 (18.2%) | 0.63 (0.33-1.2) | 0.16 |  |
| Other Hispanic | 310 (11.2%) | 0.46 (0.22-0.94) | 0.03 |  |
| Other | 206 (7.5%) | 0.44 (0.16-1.21) | 0.11 |  |
| Education level |  |  |  | 0.92 |
| < high school | 763 (27.7%) | 0.63 (0.45-0.88) | 0.006 |  |
| High school | 647 (23.5%) | 0.66 (0.44-1) | 0.05 |  |
| College | 763 (27.7%) | 0.64 (0.42-0.98) | 0.04 |  |
| > College | 581 (21.1%) | 0.62 (0.32-1.19) | 0.15 |  |
| Family income poverty ratio |  |  |  | 0.78 |
| < 1 | 817 (29.6%) | 0.56 (0.4-0.8) | 0.001 |  |
| 1–1.3 | 342 (12.4%) | 0.6 (0.36-1.02) | 0.06 |  |
| 1.3–3.0 | 786 (28.5%) | 0.65 (0.43-0.97) | 0.04 |  |
| ≥ 3.0 | 814 (29.5%) | 0.74 (0.47-1.16) | 0.19 |  |
| Diabetes |  |  |  | 0.35 |
| YES | 775 (28.1%) | 0.57 (0.42-0.77) | <0.001 |  |
| NO | 1984 (71.9%) | 0.69 (0.52-0.93) | 0.01 |  |
| Hypertension |  |  |  | 0.3 |
| YES | 2093 (75.9%) | 0.66 (0.52-0.83) | <0.001 |  |
| NO | 666 (24.1%) | 0.57 (0.36-0.91) | 0.02 |  |
| Smoke |  |  |  | 0.38 |
| Current | 582 (21.1%) | 0.8 (0.54-1.18) | 0.25 |  |
| Former | 551 (20%) | 0.54 (0.36-0.81) | 0.003 |  |
| Never | 1626 (58.9%) | 0.61 (0.44-0.84) | 0.003 |  |
| BMI |  |  |  | 0.3 |
| ≤ 25 | 657 (23.8%) | 0.52 (0.28-0.97) | 0.04 |  |
| 25–30 | 873 (31.6%) | 0.51 (0.32-0.8) | 0.003 |  |
| ≥ 30 | 1229 (44.5%) | 0.69 (0.54-0.9) | 0.005 |  |
| Oral treatment |  |  |  | 0.87 |
| YES | 473 (17.1%) | 0.69 (0.39-1.24) | 0.21 |  |
| NO | 2286 (82.9%) | 0.63 (0.5-0.79) | <0.001 |  |

**Supplementary Table 2.** Subgroup analysis on the correlation between MASLD and Inverse Simpson Index.

| Subgroup | N (%) | OR (95% CI) | *P* | *P* for interaction |
| --- | --- | --- | --- | --- |
| Age |  |  |  | 0.75 |
| 20–39 | 1282 (46.5%) | 0.06 (0.002-2.06) | 0.12 |  |
| 40–59 | 1141 (41.4%) | 0.21 (0.009-4.99) | 0.34 |  |
| ≥ 60 | 336 (12.2%) | 0.02 (0-9.64) | 0.21 |  |
| Gender |  |  | 0 | 0.18 |
| Male | 1395 (50.6%) | 0.03 (0.001-0.46) | 0.01 |  |
| Female | 1364 (49.4%) | 0.56 (0.02-19.39) | 0.75 |  |
| Race ethnicity |  |  | 0 | 1 |
| Mexican American | 597 (21.6%) | 0.08 (0.001-6.53) | 0.26 |  |
| Non-Hispanic White | 1145 (41.5%) | 0.15 (0.009-2.6) | 0.19 |  |
| Non-Hispanic Black | 501 (18.2%) | 0.19 (0-677.53) | 0.69 |  |
| Other Hispanic | 310 (11.2%) | 0.16 (0-809.753) | 0.68 |  |
| Other | 206 (7.5%) | 0.04 (0-4124.24) | 0.59 |  |
| Education level |  |  | 0 | 0.88 |
| < high school | 763 (27.7%) | 0.18 (0.003-9.33) | 0.39 |  |
| High school | 647 (23.5%) | 0.06 (0.002-2.05) | 0.12 |  |
| College | 763 (27.7%) | 0.27 (0.003-27.98) | 0.58 |  |
| > College | 581 (21.1%) | 0.04 (0-23.13) | 0.33 |  |
| Family income poverty ratio |  |  | 0 | 0.76 |
| < 1 | 817 (29.6%) | 0.13 (0.003-6.15) | 0.3 |  |
| 1–1.3 | 342 (12.4%) | 0.01 (0-1.76) | 0.08 |  |
| 1.3–3.0 | 786 (28.5%) | 0.33 (0.003-33.49) | 0.64 |  |
| ≥ 3.0 | 814 (29.5%) | 0.12 (0.002-7.14) | 0.31 |  |
| Diabetes |  |  | 0 | 0.54 |
| YES | 775 (28.1%) | 0.04 (0.001-1.02) | 0.05 |  |
| NO | 1984 (71.9%) | 0.16 (0.008-2.93) | 0.22 |  |
| Hypertension |  |  | 0 | 0.32 |
| YES | 2093 (75.9%) | 0.14 (0.01-1.95) | 0.15 |  |
| NO | 666 (24.1%) | 0.03 (0.001-1.27) | 0.07 |  |
| Smoke |  |  | 0 | 0.6 |
| Current | 582 (21.1%) | 0.14 (0.004-5.1) | 0.29 |  |
| Former | 551 (20%) | 0.01 (0-1.27) | 0.06 |  |
| Never | 1626 (58.9%) | 0.24 (0.007-8.56） | 0.44 |  |
| BMI |  |  | 0 | 0.33 |
| ≤ 25 | 657 (23.8%) | 0.03 (0-13.87） | 0.26 |  |
| 25–30 | 873 (31.6%) | 0.01 (0-0.41） | 0.01 |  |
| ≥ 30 | 1229 (44.5%) | 0.32 (0.02-5.72） | 0.44 |  |
| Oral treatment |  |  | 0 | 0.76 |
| YES | 473 (17.1%) | 0.44 (0-831.59） | 0.83 |  |
| NO | 2286 (82.9%) | 0.1 (0.01-0.91） | 0.04 |  |
